# Laser-Engineered Multilayer Coatings Based on Zinc Oxide and Lovastatin-Functionalized Bioactive Glasses for Corrosion-Resistant and Antimicrobial Stainless Steel Implants

**DOI:** 10.3390/biomimetics11040227

**Published:** 2026-03-28

**Authors:** Irina Negut, Bogdan Bita, Gabriela Dorcioman, Mihaela Dinu, Anca Constantina Parau, Carmen Ristoscu, Gratiela Gradisteanu-Pircalabioru

**Affiliations:** 1 Laser Department, National Institute for Laser, Plasma and Radiation Physics, 409 Atomistilor Street, Magurele, 077125 Ilfov, Romania; negut.irina@inflpr.ro (I.N.); gabriela.dorcioman@inflpr.ro (G.D.); 2 National Institute for Research and Development in Optoelectronics INOE 2000, Magurele, 077125 Ilfov, Romania; bogdan.bita@inoe.ro (B.B.); mihaela.dinu@inoe.ro (M.D.); anca.parau@inoe.ro (A.C.P.); 3 eBio-Hub Research Center, University Politehnica of Bucharest—CAMPUS, 6 Iuliu Maniu Boulevard, 061344 Bucharest, Romania; gratiela.gradisteanu@icub.unibuc.ro; 4 Faculty of Biology and the Research Institute of the University of Bucharest (ICUB), University of Bucharest, 050657 Bucharest, Romania

**Keywords:** bioactive glass, lovastatin, zinc oxide, laser-assisted deposition, antimicrobial coatings

## Abstract

Stainless steel (SS) remains widely used in orthopedic implants but is susceptible to corrosion and implant-associated infections in physiological environments. This study aimed to develop a multifunctional multilayer coating combining corrosion resistance, bioactivity, and antimicrobial performance. A ZnO base layer was deposited on 316L SS via pulsed laser deposition, followed by matrix-assisted pulsed laser evaporation of a lovastatin-functionalized bioactive glass (BG57 + LOV) top layer. Two LOV concentrations were initially evaluated, and BG57+0.1LOV was selected based on structural homogeneity, cytocompatibility, and antimicrobial balance. Physicochemical characterization confirmed preservation of chemical integrity and formation of continuous, moderately rough coatings. Electrochemical impedance spectroscopy in simulated body fluid demonstrated progressive improvement in corrosion resistance from bare SS to ZnO-coated and finally to the BG57+0.1LOV/ZnO multilayer, which exhibited the most electropositive corrosion potential and effective suppression of charge-transfer reactions. Biological assays revealed high viability of osteoblasts, fibroblasts, keratinocytes, and macrophages without significant oxidative or nitrosative stress. Antimicrobial testing showed strain-dependent activity, with enhanced efficacy against MRSA and significant reduction in *P. aeruginosa*, associated with increased ROS/RNS generation. Overall, the BG57+0.1LOV/ZnO system represents a promising multifunctional coating strategy for corrosion-resistant and infection-resistant SS implants.

## 1. Introduction

Stainless steel (SS), particularly 316L, remains one of the most widely used metallic biomaterials in orthopedic and temporary implant applications due to its favorable mechanical strength, cost-effectiveness, and ease of processing [1]. However, despite its broad clinical use, SS is susceptible to corrosion in physiological environments [2]. The presence of chloride ions in body fluids can destabilize the passive oxide layer formed on SS, leading to pitting corrosion, ion release, and potential inflammatory responses [3]. Long-term electrochemical degradation may compromise implant stability and contribute to biological complications [4]. Consequently, improving the corrosion resistance of SS through surface modification remains a priority in biomaterials research.

In parallel, implant-associated infections and corrosion-related degradation remain critical challenges in orthopedic and biomedical implantology [5,6]. Microbial colonization of implant surfaces can rapidly lead to biofilm formation, chronic inflammation, and implant failure, while corrosion processes in physiological environments compromise mechanical stability and release potentially harmful degradation products [7]. Recent clinical analyses emphasize that biomaterial-associated infections remain among the leading causes of revision surgery in orthopedic practice [7,8]. Therefore, the development of multifunctional surface coatings capable of simultaneously improving corrosion resistance and preventing microbial colonization is of high biomedical relevance [9]. Simultaneously, corrosion processes in body fluids are recognized as key factors influencing long-term implant stability and tissue response [10].

Zinc oxide (ZnO) has emerged as a promising candidate for implant surface modification due to its intrinsic antimicrobial activity [11] and corrosion protection capability [12,13]. ZnO-based coatings exert antibacterial effects primarily through the generation of reactive oxygen species (ROS), membrane disruption, and zinc ion release, which collectively induce oxidative stress and cellular damage in bacteria [14]. Beyond antimicrobial effects, Zn^2+^ ions also contribute to osteogenic activity, supporting bone cell proliferation and mineralization [15]. More broadly, ZnO nanostructured coatings have been widely reported to provide strong antimicrobial activity and can be integrated into composite biomaterial coatings for implant surface functionalization [16].

Bioactive glasses (BG), including compositions such as BG57 [17], are widely recognized for their osteoconductive and osteoinductive properties. Upon immersion in physiological fluids, BG promote hydroxyapatite formation and stimulate bone regeneration through controlled ionic dissolution [18]. Recent developments highlight their ability to modulate cellular responses and improve tissue integration while maintaining degradability [19]. Nevertheless, although BGs promote bone bonding and tissue regeneration, their intrinsic antibacterial effect is generally limited and often insufficient to prevent implant-associated infections and biofilm formation/development [17]. Several studies emphasize that conventional BGs often require incorporation of antibacterial ions or therapeutic agents to achieve effective and sustained anti-biofilm performance [20]. Metallic ion doping remains a dominant approach, including, e.g., silver [21], cerium [22], zinc [23], and tantalum [24] to impart antimicrobial, pro-angiogenic, and osteogenic properties. In parallel, antibiotic-integrated BG systems, such as gentamicin-loaded borosilicate BGs, have been developed to synergistically combat infection while promoting osseointegration [25]. Moreover, multifunctional hybrid systems combining drug-loaded and ion-doped mesoporous BG structures have been proposed to simultaneously support hemostasis, angiogenesis, and antimicrobial activity [26].

Combined systems based on Zn-doped mesoporous BGs loaded with antibiotics such as vancomycin or levofloxacin have demonstrated synergistic antibacterial effects against pathogens including *Staphylococcus aureus* and *Escherichia coli*, while also supporting tissue regeneration [27]. Additionally, ZnO-containing BG have shown antimicrobial activity against pathogenic bacteria, highlighting the potential of Zn-modified BGs for infection-resistant biomedical coatings [28].

Lovastatin (LOV), a well-known HMG-CoA (hydroxymethylglutaryl-coenzyme A) reductase inhibitor, has attracted attention in regenerative medicine due to its osteogenic and immunomodulatory effects [29]. Beyond lipid-lowering activity, LOV stimulates bone morphogenetic protein-2 (BMP-2) expression and enhances osteoblastic differentiation [30]. Furthermore, recent studies indicate that statins exhibit antimicrobial properties, particularly against Gram-positive bacteria, and can interfere with microbial membrane-associated metabolic pathways [14]. Additionally, statins have demonstrated antifungal effects through disruption of ergosterol biosynthesis in *Candida* species [31]. These findings suggest that lovastatin incorporation into bioactive coatings may confer dual osteogenic and antimicrobial benefits [32,33].

While LOV has been incorporated into various polymeric and calcium phosphate-based scaffolds to enhance osteogenesis [34,35], statin-functionalized implant coatings have demonstrated antibiofilm and osteogenic potential [36]. However, reports on BG + LOV systems remain very limited.

While extrusion-, inkjet-, and light-based 3D bioprinting dominate current biofabrication workflows, laser-based deposition techniques such as pulsed laser deposition (PLD) and matrix-assisted pulsed laser evaporation (MAPLE) are increasingly recognized as powerful, complementary approaches for the precision biofunctionalization of printed constructs. These techniques enable the controlled transfer of inorganic, organic, and hybrid biomaterials with sub-micrometer resolution, offering capabilities that extend beyond conventional nozzle-based printing [37,38].

Laser-assisted biofabrication methods, including MAPLE, have been shown to allow the gentle deposition of fragile biomolecules, polymers, and nanocomposites while preserving their chemical structure and biological functionality, owing to cryogenic target conditions and minimal thermal load [39].

In particular, laser deposition has emerged as a viable technique to improve the surface properties of different scaffolds fabricated by additive manufacturing [40,41,42]. Recent studies demonstrate that hybrid biofabrication strategies combining 3D printing and MAPLE enable hierarchical scaffold design, where macroscopic architecture is defined by additive manufacturing, while nanoscale surface chemistry and bioactivity are introduced via laser deposition [43]. Such approaches have proven effective in enhancing cell adhesion, osteogenic differentiation, and biological performance without compromising mechanical integrity.

Similarly, PLD offers exceptional control over thin-film composition, crystallinity, and thickness, making it particularly valuable for the deposition of bioactive ceramic layers, antibacterial coatings, and functional oxide films on biomedical substrates. PLD-fabricated coatings are widely used to modulate surface bioactivity, corrosion resistance, and cell–material interactions in implantable systems [44].

From a broader perspective, laser-based deposition techniques align closely with emerging trends in hybrid and multimodal biofabrication, where printing, coating, and in situ functionalization are combined to produce smart, bioactive, and clinically translatable constructs [37,38]. These technologies are particularly relevant for applications beyond classical tissue engineering, including implantable therapeutic platforms, biohybrid systems, and advanced in vitro models, where precise control over surface chemistry and biointerface design is critical [45].

Multilayer functional coatings provide a design advantage over single-layer BG coatings because they enable functional compartmentalization: a bottom layer can be optimized for adhesion, corrosion protection, and mechanical stability, while the top layer(s) can be tailored for bioactivity and controlled antimicrobial/drug release. Reported multilayer BG systems demonstrate this principle. Consequently, multilayer or hierarchical coating strategies are increasingly explored to decouple biological activity from corrosion protection and achieve controlled therapeutic delivery [46,47]. For example, layered PEEK/BG architectures have been used as a stable base layer with a bioactive/antibacterial top layer containing an antimicrobial molecule such as lawsone, yielding bioactivity together with antibacterial performance and improved corrosion resistance [48]. More generally, stratified (multilayer) composite coatings have been shown to mitigate drawbacks of single-layer systems by controlling antimicrobial ion release: a bilayer design with a BG-containing base and a thin antibacterial top layer enabled potent antibacterial action while maintaining osteoblast compatibility, whereas comparable single-layer coatings exhibited burst release and cytotoxicity [49]. Multilayer concepts are also being applied in advanced implant platforms, including polyelectrolyte multilayers incorporating mesoporous BG doped with therapeutic ions to support osseointegration/angiogenesis and sustained antibacterial effects [50].

In our research group, we previously developed multilayer coatings incorporating BG and therapeutic agents (e.g., ciprofloxacin, doxycycline, neem, holy basil and turmeric) onto polymeric base layers (e.g., polymethylmethacrylate (PMMA)), using laser-assisted deposition techniques [18,51,52,53]. Those systems demonstrated improved biocompatibility and controlled degradation profiles; also, polymeric interlayers such as PMMA provided corrosion protection for metallic substrates [18,51,52,53].

These reports support the rationale that multilayer coatings can outperform single-layer BG coatings by improving long-term interfacial stability while enabling tunable, safer antimicrobial delivery and maintaining bioactivity. This approach is aligned with the present work on multilayer functional coating strategy.

Despite the individual advantages of ZnO, BG, and LOV, multilayer systems integrating a ZnO corrosion-resistant base layer with a LOV-functionalized BG top layer remain insufficiently explored. In this context, the novelty of the present work is to move beyond “single-material, single-layer multifunctionality” by using a multilayer functional architecture that spatially separates (i) corrosion protection/barrier function from (ii) ion-driven bioactivity and (iii) antimicrobial/therapeutic delivery, thereby aiming to decouple competing requirements that are difficult to satisfy simultaneously in a single layer.

Therefore, the aim of this study was to design and evaluate multifunctional BG57 + LOV/ZnO multilayer coatings combining corrosion resistance, cytocompatibility, and antimicrobial activity. The ZnO layer was deposited on SS via PLD to provide interfacial protection, while the LOV-loaded BG57 layer was fabricated using MAPLE to preserve the structural integrity of the bioactive components. A comprehensive physicochemical, electrochemical, biological, and antimicrobial assessment was performed to determine whether this multilayer strategy offers a balanced and synergistic solution for next-generation biomedical implant coatings.

## 2. Materials and Methods

### 2.1. Materials

Dimethyl sulfoxide (DMSO, ≥99.9%), analytical-grade acetone (≥99.5%), and ethanol (≥99.8%) were used. All chemicals required for the preparation of simulated body fluid (SBF), namely NaCl (≥99.5%), NaHCO_3_ (≥99.7%), KCl (≥99.5%), K_2_HPO_4_·3H_2_O (≥99%), MgCl_2_·6H_2_O (≥98%), HCl (37%, analytical grade), CaCl_2_ (≥96%), Na_2_SO_4_ (≥99%), and tris(hydroxymethyl)aminomethane ((CH_2_OH)_3_CNH_2_, ≥99.8%) were supplied by Sigma-Aldrich Chemie GmbH (Steinheim, Germany).

Lovastatin (LOV) (99.99%) was purchased from Selleck Biotechnology GmbH (Koln, Germany).

A commercially available ZnO (99.999%) target purchased from Kurt J. Lesker Company GmbH (Dresden, Germany) was employed for the PLD experiments.

The SBF, designed to reproduce the ionic composition of human blood plasma, was prepared according to a modified Kokubo protocol by sequentially dissolving the reagents in deionized water using the prescribed order and concentrations [54].

Bioactive glass powders (BG57) were synthesized following previously reported procedures. The BG57 composition belongs to the multicomponent SiO_2_–Na_2_O–K_2_O–CaO–MgO–P_2_O_5_ glass system.

Next, 316L SS and Si (100) substrates with surface areas of (1 × 1) cm^2^ were employed for thin film deposition. Prior to deposition, substrates underwent a standardized cleaning procedure to remove surface contaminants. This protocol consisted of sequential ultrasonic treatment for 15 min in acetone, ethanol, and deionized water, using an ultrasonic bath (Elma Schmidbauer GmbH, Singen, Germany). After cleaning, the substrates were dried under a stream of high-purity nitrogen gas (N_2_, purity 5.0). The prepared substrates were then fixed onto a sample holder and transferred into the deposition chamber for further processing.

### 2.2. Laser Deposition of Thin Films

Three coating configurations were fabricated on SS substrates by combining PLD and MAPLE as follows:

(i) ZnO base-layer synthesized onto SS by PLD

In our previous studies, we developed multilayer coatings incorporating bioactive glass and therapeutic agents onto PMMA base layers, deposited via the MAPLE technique, where PMMA served as a corrosion-protective interlayer [18,51,52,53]. Although these systems demonstrated improved cytocompatibility and controlled drug release, the MAPLE process requires dissolution of the material into a suitable solvent prior to deposition [55]. In contrast, the ZnO interlayer used in the present work was fabricated by PLD, a technique that enables direct ablation of a solid target without the need for solubilization [56]. This solvent-free approach simplifies processing, avoids potential solvent-related effects, and allows precise control of inorganic thin-film growth, making ZnO a more robust and technologically straightforward corrosion-protective base layer for metallic substrates.

ZnO thin films were deposited onto SS and Si (100) substrates by PLD technique in a vacuum chamber. A KrF* excimer laser (λ = 248 nm, τ_FWHM_ = 25 ns; COMPexPro 205F, Coherent) was employed as an ablation source, operating at a repetition rate of 5 Hz. During deposition, the chamber was evacuated and maintained at a residual pressure of 10^−5^ mbar. The ZnO target was continuously rotated at a constant speed (50 rpm) to prevent target drilling and to ensure uniform ablation. The laser beam was focused onto the target surface at an incidence angle of 45°. ZnO films were deposited using pulse energy of 350 mJ, delivered over an irradiated area of 10 mm^2^, with a total of 1000 laser pulses applied for each deposition.

(ii) BG57 + LOV single layer deposited on bare SS by MAPLE

BG57 and LOV (BG57 + LOV) dissolved in DMSO served as solution for MAPLE targets. 0.01 or 0.005 g of LOV in DMSO were mixed using a magnetic stirrer with 0.02 g of BG57and were further denoted as BG57 + 0.1LOV and BG57 + 0.5LOV, respectively. The prepared solutions were cast onto a copper support and rapidly frozen in liquid nitrogen to obtain solid cryogenic targets. These frozen targets were mounted on a temperature-controlled holder inside the vacuum deposition chamber and maintained at liquid nitrogen temperature throughout the MAPLE process. To ensure homogeneous ablation and to minimize localized thermal or mechanical damage, the frozen target was continuously rotated at 50 rpm during laser irradiation.

(iii) Bi-layered structure BG57 + LOV/ZnO, in which the BG57 formulation containing the optimal LOV concentration was applied by MAPLE onto a pre-existing ZnO layer obtained by PLD. For this stage, BG57 + 0.1LOV was selected. The same procedure and laser parameters as in step (ii) were used.

### 2.3. Thin-Film Characterization Methods

#### 2.3.1. Scanning Electron Microscopy and Elemental Analysis

The surface morphology of the thin films was examined using a high-resolution scanning electron microscope (Apreo S, Thermo Fisher Scientific, Hillsboro, CA, USA) (SEM), offering a spatial resolution of up to 0.7 nm. SS coated substrates were selected for top-view surface analysis. For cross-section analyses, we chose the Si substrates. SEM observations were conducted at an accelerating voltage of 10 kV under a high-vacuum environment of approximately 1 × 10^−3^ Pa. To mitigate charging effects during electron beam exposure, all specimens were coated with a thin conductive gold layer prior to analysis. Elemental composition of the deposited coatings was further investigated using the same SEM system equipped with a silicon–lithium (SiLi) energy-dispersive X-ray spectroscopy (EDX) detector.

#### 2.3.2. Atomic Force Microscopy

The surface topography of the ZnO coatings was additionally analyzed by atomic force microscopy (AFM) with an Inova model (Veeco/Bruker, Billerica, MA, USA) operated in tapping mode. AFM measurements for ZnO and BG57 + LOV thin films were performed using a Veeco apparatus over a surface area of 10 µm^2^, at a scanning rate of 0.3 Hz and an image resolution of 512 pixels. An RTESPA probe was used for all measurements. For BG57 + LOV/ZnO we used the same apparatus over a surface area of 5 µm^2^, at a scanning rate of 0.5 Hz and an image resolution of 512 pixels.

#### 2.3.3. Fourier Transform Infrared Spectroscopy

Fourier transform infrared (FTIR) spectroscopy was employed to investigate chemical modifications of the samples. Spectral analyses were carried out using a Shimadzu IRTracer-100 spectrometer (Waltham, MA, USA) operating in absorbance mode over the 8000–400 cm^−1^ wavenumber range, with a spectral resolution of 4 cm^−1^. Data acquisition was performed using an attenuated total reflectance (ATR) accessory, and each spectrum represented the average of 92 accumulated scans to ensure an adequate signal-to-noise ratio.

#### 2.3.4. Electrochemical Behavior of Samples

The electrochemical behavior of the ZnO layer, BG57 + LOV coating, and the BG57 + 0.1LOV/ZnO bi-layer system was investigated using Electrochemical Impedance Spectroscopy (EIS). Measurements were performed in SBF at 37 °C using a VersaSTAT 3 potentiostat (Princeton Applied Research, Oak Ridge, TN, USA).

A sinusoidal perturbation signal of 10 mV RMS was applied versus the open circuit potential (EOC) over a frequency range of 0.1–10^4^ Hz (for BG-based coatings) and 0.25–10^3^ Hz (for ZnO single-layer samples). A conventional three-electrode corrosion cell was employed, consisting of a saturated Ag/AgCl reference electrode (0.197 V vs. SHE), a platinum counter electrode, and the coated sample as the working electrode.

For ZnO-coated samples, the specimens were immersed in SBF for 24 h, and EIS spectra were recorded after 6, 12, 18, and 24 h to evaluate time-dependent degradation behavior. For BG-based coatings, the open circuit potential was monitored for 1 h following immersion in SBF at 37 °C to assess the stabilization of the material–electrolyte interface and the formation of a protective passive layer.

EIS data were acquired using VersaStudio software (version 2.60.6, Princeton Applied Research, Oak Ridge, TN, USA), while equivalent circuit fitting and parameter extraction were performed using ZView (version 12136-4, Scribner Associates Inc., Southern Pines, NC, USA). The obtained parameters provide insight into the interfacial processes, coating stability, passive layer formation, and corrosion resistance of the investigated systems. 

#### 2.3.5. The Surface Wettability of the Samples

The wettability of the MAPLE-deposited coatings evaluated their hydrophilic or hydrophobic character. The wettability of the coatings was assessed using the sessile drop technique, employing an Attension Theta Lite (TL) 101 optical tensiometer (version 1.0.3, Biolin Scientific, Vastra Frolunda, Sweden). This evaluation was performed under atmospheric conditions, specifically at a temperature of 22 ± 1 °C and a relative humidity of 42%. During the analysis, the contact angle (CA) between the test liquid and the surface of the specimens was precisely measured. Droplets of SBF were gently placed on the coating surfaces, and the resulting CA was recorded to assess surface affinity toward aqueous environments.

#### 2.3.6. Biological Evaluation of MAPLE-Coated Samples

##### Biocompatibility Assays

The biocompatibility of BG57 + LOV single-layers deposited on bare SS by MAPLE was evaluated using G292 osteoblast cells. The cells were cultured in Clonetics™ OGM™ Osteoblast Growth Medium for 24 h at 37 °C, under 95% humidity and 5% CO_2_. Subsequently, the cells were washed with saline solution (Sigma Aldrich Chemie GmbH, Steinheim, Germany), trypsinized (0.25% trypsin-EDTA, Thermo Scientific, Hillsboro, CA, USA), and counted using Trypan Blue and a hemocytometer. The substrates were co-cultured with the cells at a seeding density of 5 × 10^5^ cells/well for 24 h (37 °C, 95% humidity, 5% CO_2_).

For BG57 + LOV single layers deposited on bare SS by MAPLE, cell viability and proliferation were evaluated using the MTT assay (3-(4,5-dimethylthiazol-2-yl)-2,5-diphenyltetrazolium bromide), a quantitative method for assessing metabolically active cells in culture. The MTT compound is permeable to viable cell membranes and, following mitochondrial metabolism, is reduced to purple formazan crystals. After incubation for 4 h at 37 °C under 95% humidity and 5% CO_2_ (Vybrant^®^ MTT Cell Proliferation Assay Kit, Thermo Scientific, Hillsboro, CA, USA, cat. no. V-13154), the formed formazan crystals were solubilized using SDS-HCl for 18 h under identical conditions. The resulting purple solution was measured at 550 nm using a Multiskan FC spectrophotometer (Thermo Scientific, Hillsboro, CA, USA), and absorbance values were directly correlated with the number of viable cells.

For the BG57 + LOV/ZnO bi-layer system, biocompatibility was further evaluated on three cell lines: human dermal fibroblasts (HDF), epithelial cells (HaCaT), and monocytes (RAW). Cells were cultured in DMEM supplemented with 10% fetal bovine serum and seeded at a density of 1 × 10^4^ cells per well. After 24 h of incubation with the tested materials, the culture medium was removed and the surfaces were washed with PBS to eliminate residual serum that could interfere with the MTT reagent. Each sample was then incubated with 1 mL of MTT solution (1 mg/mL) for 4 h at 37 °C and 5% CO_2_. The resulting formazan crystals were dissolved in isopropanol, and the optical density of the violet solution was measured at 550 nm. The intensity of the absorbance signal was directly proportional to the number of viable and metabolically active cells.

All BG + LOV coatings cytotoxicity was further evaluated using the Lactate Dehydrogenase (LDH) Cytotoxicity Detection Kit (Roche). LDH activity was measured in the supernatant using a Multiskan FC instrument (Thermo Scientific) at λ = 490 nm, with a reference wavelength of λ = 600 nm. Cells that lose membrane integrity release cytoplasmic LDH into the culture medium; therefore, LDH quantification provides a quantitative measure of cell death. For LDH determination, 100 µL of reaction mixture was prepared, containing equal proportions of all assay components. From each tested sample, 50 µL of culture medium was collected in duplicate and transferred into a 96-well plate. Subsequently, 100 µL of the reaction mix was added to each well, and the plate was incubated for 15–20 min in the dark. The enzymatic reaction produces a pink-colored solution, whose intensity is directly proportional to the amount of LDH released and, consequently, to the number of dead cells. Absorbance was measured spectrophotometrically at 490 nm using a Tecan Infinite 200Pro plate reader (GENios Tecan, Ramsey, MN, USA).

For a sample to be considered biocompatible, the optical density values obtained from the MTT assay must be higher than those from the LDH quantification test. More specifically, a sample is regarded as biocompatible if the number of viable and metabolically active cells exceeds the number of dead cells. The MTT assay measures mitochondrial metabolic activity and is therefore an indicator of cellular viability.

The evaluation of osteoblast biocompatibility also included the Live/Dead assay. This qualitative test uses calcein, a green fluorescent compound that is permeable only to viable cells and stains them green, and ethidium bromide, an intercalating agent that binds to nucleic acids and stains dead cells red. The assessment of cells G292 co-cultured onto BG + LOV thin films using the Live/Dead assay was performed with a Zeiss Axioscope fluorescence microscope (Oberkochen, Germany). The biocompatibility of the BG57 + LOV/ZnO coatings was evaluated using HDF and HaCat cells. Cells were cultured in DMEM supplemented with 10% fetal bovine serum and seeded at a density of 1 × 10^4^ cells per well in 750 µL of culture medium. The cells were incubated together with the synthesized materials for 3 days at 37 °C under 5% CO_2_. The Live/Dead staining protocol was performed as follows: after incubation, the culture medium was removed and the cell layer was gently washed with PBS. A Live/Dead staining solution containing calcein-AM and ethidium bromide was prepared and 500 µL of the solution was added to each well containing the tested materials. The plate was incubated for 1 h in the dark. Fluorescence imaging was subsequently carried out using a Zeiss Axioscope microscope (Oberkochen, Germany) to evaluate cell viability and morphology.

Cytotoxicity and cell viability tests (MTT, LDH, and Live/Dead assays) were performed in accordance with ISO 10993-5:2009 (Biological evaluation of medical devices—tests for in vitro cytotoxicity) and ISO 10993-12:2021 (sample preparation and reference materials).

The level of reactive oxygen species (ROS) induced by the tested materials was quantified. For this purpose, RAW macrophages (1 × 10^6^ cells/well) were stimulated for 2 h with the materials in phenol red-free RPMI medium. Hydrogen peroxide production was quantified using the 2′,7′-dichlorofluorescein diacetate (DCFDA) assay kit, according to the manufacturer’s instructions. The results were expressed as relative fluorescence units (RFU).

Reactive nitrogen species (RNS) quantification was performed using the Nitric Oxide Assay Kit (Thermo Scientific), based on the Griess reaction. The test was conducted on osteoblasts after 24 h of incubation with the investigated biomaterials.

##### Antimicrobial Activity

The antimicrobial activity of the synthesized thin films was investigated using standard microbial strains: *Staphylococcus aureus* ATCC 25923, *Escherichia coli* ATCC 25922, *Pseudomonas aeruginosa* ATCC 27853, and *Candida albicans* ATCC 26790, *E. faecalis*, methicillin -resistant *Staphylococcus aureus (MRSA).*

The anti-biofilm activity of BG57-based thin films was evaluated following UV sterilization of the samples. Primary microbial cultures were diluted 1:100 in fresh medium to obtain an optical density of approximately OD_600nm_ ≈ 0.01. Subsequently, 5 mL of the diluted microbial suspension was added to tubes containing the biomaterials to be tested. The samples were half-immersed to promote biofilm formation at the air–liquid interface.

The tubes were tightly closed and incubated at 37 °C under mild agitation (50 rpm) for 72 h. After incubation, the BG-based samples were carefully separated from planktonic non-adherent cells and culture tubes.

Biofilm formation was quantified using crystal violet staining. Following three washes with autoclaved ultrapure water, the plates were stained with 0.1% crystal violet solution for 10–15 min, followed by three additional washing steps. The stained biofilms were air-dried and subsequently solubilized in 33% acetic acid for 15–20 min. The solubilized fractions were transferred in triplicate (100 μL each) into 96-well plates, and absorbance was measured at 550 nm using a Tecan Pro UV–Vis spectrophotometer (Tecan, Ramsey, MN, USA).

To determine the influence of the tested materials (BG57 + LOV/ZnO) on microbial viability, the samples were first sterilized by UV exposure (30 min on each side) and then brought into contact with microbial suspensions at a density of 10^6^ CFU/mL (colony-forming units per milliliter) in a liquid medium that maintains bacterial viability without allowing proliferation. After 24 h of incubation, microbial viability was assessed by performing serial dilutions followed by plating on agar media for CFU counting.

The antibacterial activity evaluation was performed following commonly used microbiological procedures based on ISO 22196:2011 https://www.iso.org/standard/54431.html (measurement of antibacterial activity on plastics and other non-porous surfaces), adapted for metallic substrates.

## 3. Results

### 3.1. ZnO Base-Layer Synthesized onto SS by PLD

**Figure 1:**
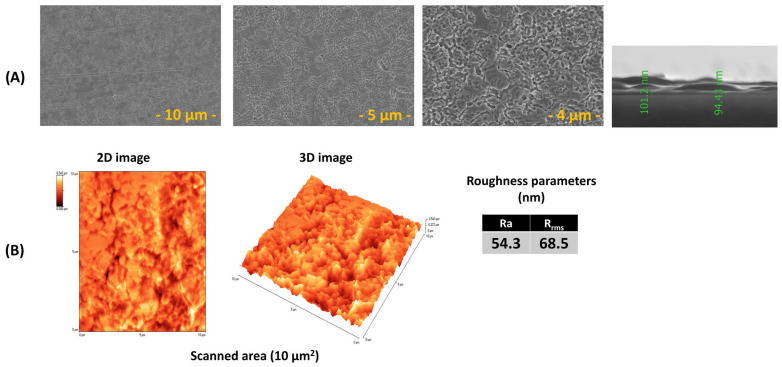
(**A**) SEM and (**B**) AFM images, and the corresponding surface roughness parameters of ZnO thin films obtained by PLD.

Figure 1 presents top-view images acquired at different magnifications and cross-section SEM micrographs (A), AFM topography images (B), and the corresponding surface roughness parameters of PLD ZnO thin films.

A heterogeneous surface texture was observed for the films, characterized by a root mean square roughness (Rrms) of about 68.5 nm. This level of roughness corroborates the morphological features identified by SEM analysis.

**Figure 2:**
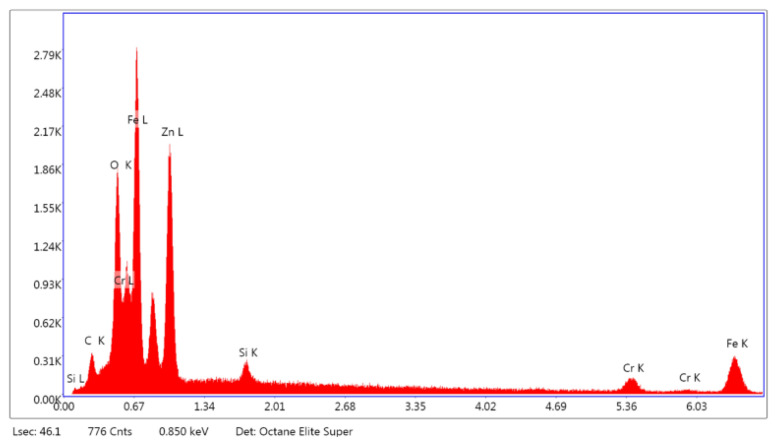
EDS spectrum of the ZnO thin film.

The specific chemical elements of the obtained ZnO coating and SS were identified by EDS (Figure 2).

Fe and Cr signals correspond to the SS substrate. The pronounced Zn peak(s) and the concurrent O signal indicate the formation of a Zn–O-based surface layer consistent with ZnO (Figure 2). Notably, the O peak is not unique to ZnO because SS also contains O in its passive Cr-rich oxide.

**Figure 3:**
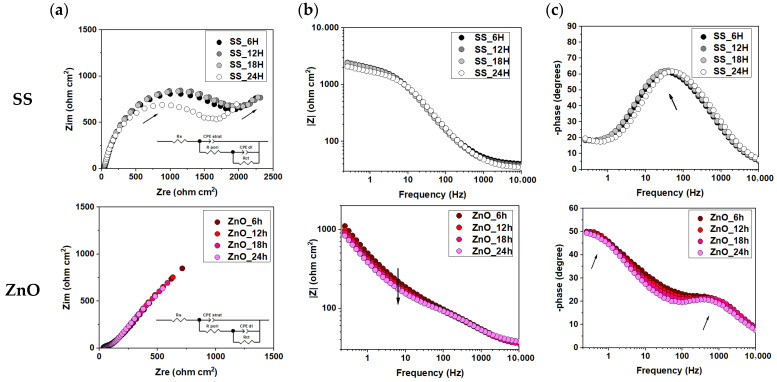
Electrochemical impedance spectra of the bare SS and ZnO coating: (**a**) Nyquist representation and Bode plots of (**b**) impedance magnitude and (**c**) phase angle.

Figure 3 shows the impedance spectra obtained for bare SS substrates as compared with ZnO thin films deposited on SS substrates, while the electrochemical parameters extracted from the fitting procedure are summarized in Table 1.

### 3.2. BG57 + LOV Single-Layers Deposited on Bare SS by MAPLE

**Figure 4:**
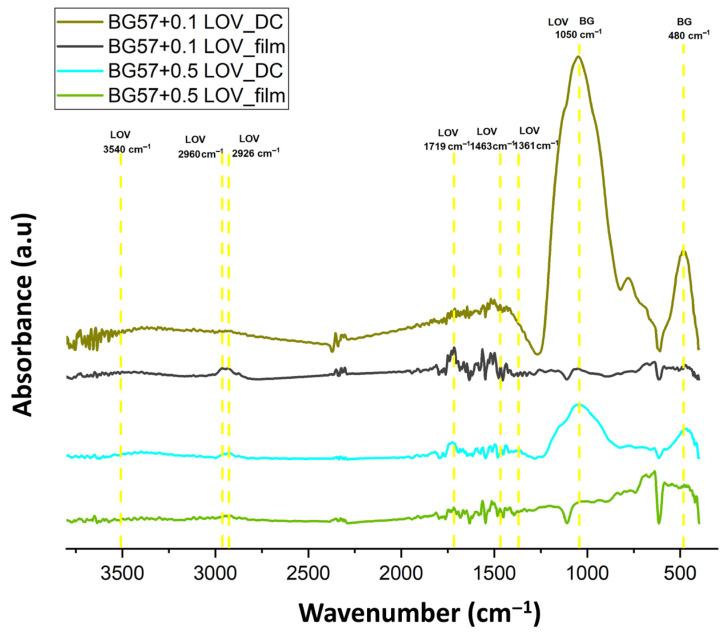
FTIR spectra of BG + LOV/SS thin films.

FTIR was used to evaluate the stoichiometry and chemical integrity of the thin films after MAPLE deposition in comparison with the drop-cast samples, assessing their potential compositional changes (Figure 4).

**Figure 5:**
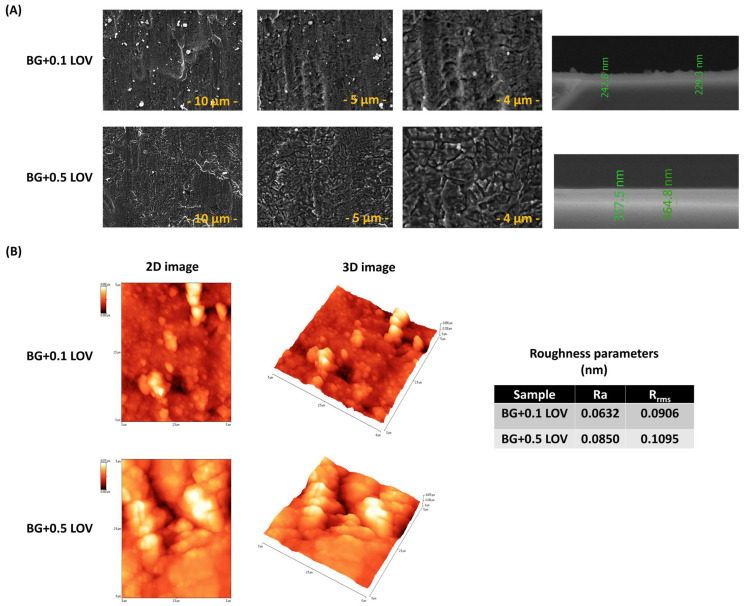
Top-view SEM images recorded at different magnifications along with corresponding cross-sectional SEM micrographs (**A**), AFM topography images, and the corresponding surface roughness parameters (**B**) of the BG + LOV thin films.

As shown in Figure 5, the surface morphology of the BG + LOV thin films was characterized by SEM at different magnifications (A), complemented by AFM topographical analysis and the corresponding roughness parameters (B).

**Figure 6:**
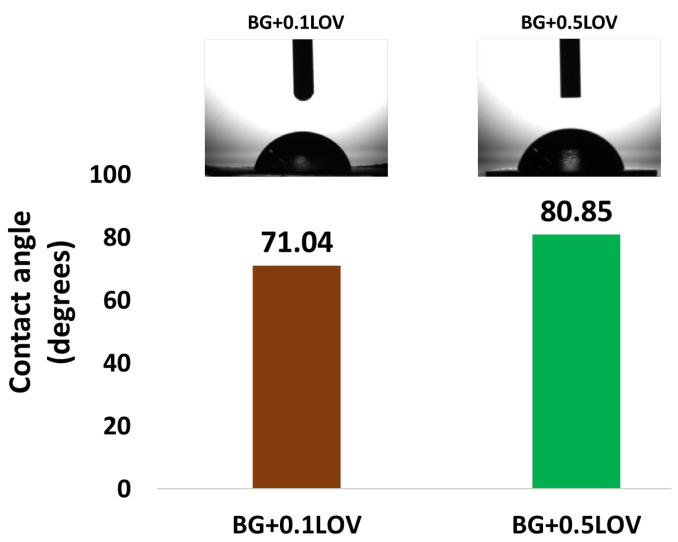
CA of investigated samples.

To evaluate the wettability of the obtained thin films, the CA between the sample surfaces (BG + 0.1 LOV and BG + 0.5 LOV) and SBF was measured (Figure 6).

**Figure 7:**
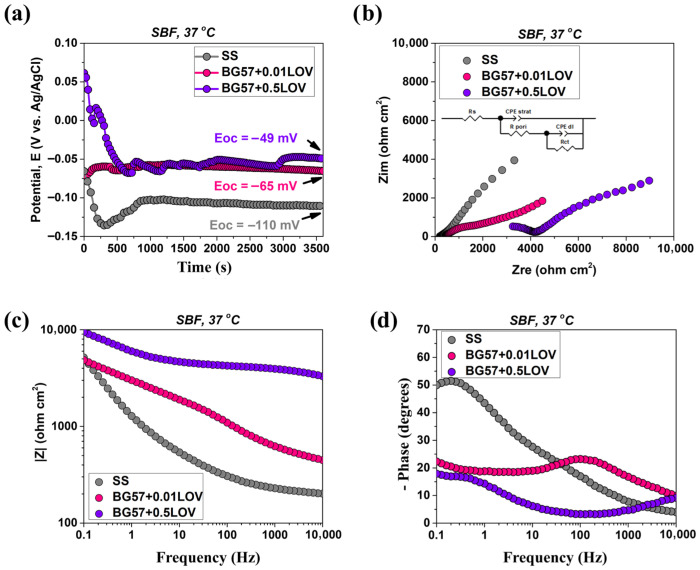
(**a**) Evolution of the open circuit potential over time, (**b**) Nyquist diagram, (**c**) Bode amplitude diagram and (**d**) Bode phase diagram for BG57-based coating deposited onto bare SS.

Figure 7a shows the evolution of the open circuit potential over time, with values obtained in the absence of potential perturbations. This method evaluates the electrochemical state of the surface and its tendency to form a stable protective layer. Therefore, the evolution of Eoc was analyzed to gain insight into the changes occurring as a result of immersion in SBF (37 °C). The Nyquist, Bode amplitude and phase EIS diagrams are further presented in Figure 7b–d, respectively.

The electrochemical parameters determined from the interpretation of the impedance curves obtained after 1 h of immersion in SBF (37 °C) are presented in Table 2.

**Figure 8:**
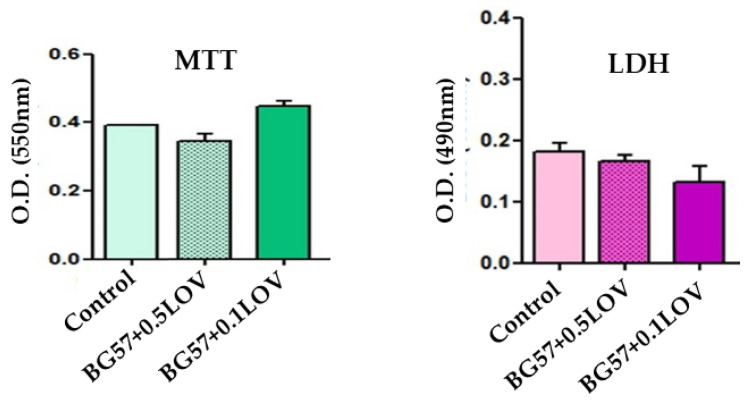
Thin films biocompatibility using MTT (**left**) and LDH (**right**) assays.

The in vitro biocompatibility of the BG57-based thin films incorporating LOV was evaluated using MTT and LDH assays after 24 h of incubation with G292 osteoblasts (Figure 8).

**Figure 9:**
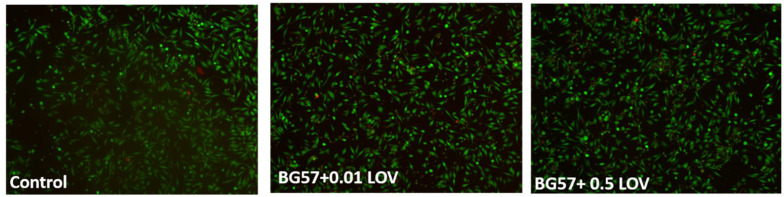
Live/dead test made on MAPLE-obtained thin films. Magnification bar: 100 µm.

Figure 9 illustrates the qualitative Live/Dead staining of G292 osteoblasts after 24 h of co-culture with the BG-based thin films.

**Figure 10:**
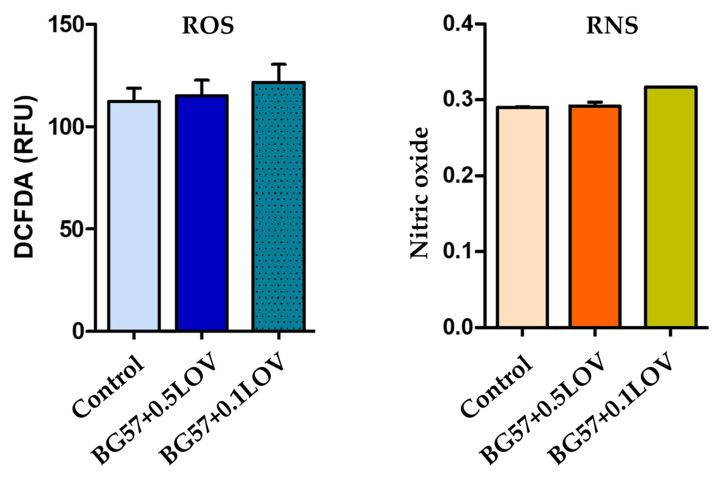
Evaluation of oxidative stress: quantification of hydrogen peroxide production using the DCFDA assay (**left**) and quantification of nitric oxide production using the Griess reaction (**right**).

To further assess the biological safety of the developed BG57-based thin films, oxidative stress markers were evaluated by quantifying both ROS and RNS. Figure 10 presents the results of hydrogen peroxide production measured using the DCFDA assay (left) and nitric oxide production determined through the Griess reaction (right). These assays provide complementary information regarding the potential of the investigated materials to induce oxidative or nitrosative stress in exposed cells, which is a critical parameter for their suitability in biomedical applications.

**Figure 11:**
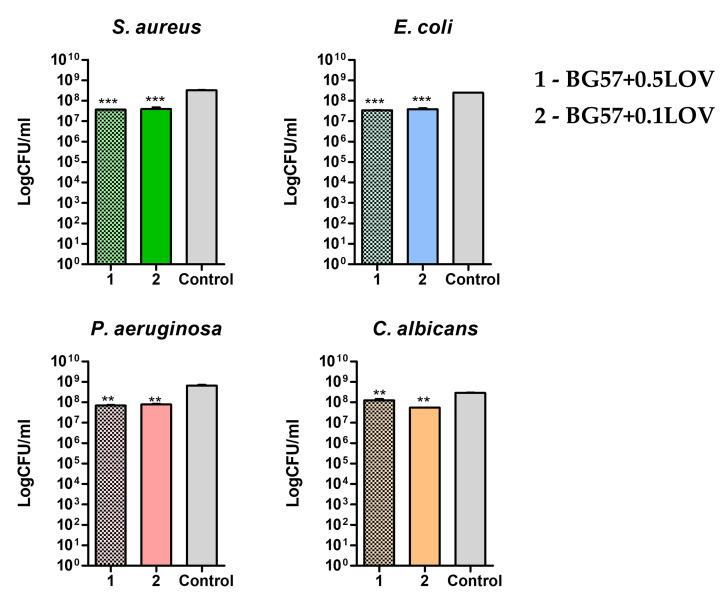
Effect of the developed materials on the viability of *S. aureus*, *E. coli*, *P. aeruginosa*, *C. albicans*.

The antimicrobial effect of the developed materials against *S. aureus*, *E. coli*, *P. aeruginosa*, *C. albicans* infectious microorganisms (Figure 11) was evaluated by assessing bacterial viability following exposure to the tested coatings. The results provide insight into the ability of the biomaterial surfaces to inhibit bacterial survival and reduce the risk of implant-associated infections.

### 3.3. BG57 + LOV/ZnO Bi-Layered Structure on SS

Preliminary investigations on structural stability, biological performance, and antimicrobial efficacy lead to the selection of BG + 0.1LOV to be applied on SS substrates previously coated with ZnO.

**Figure 12:**
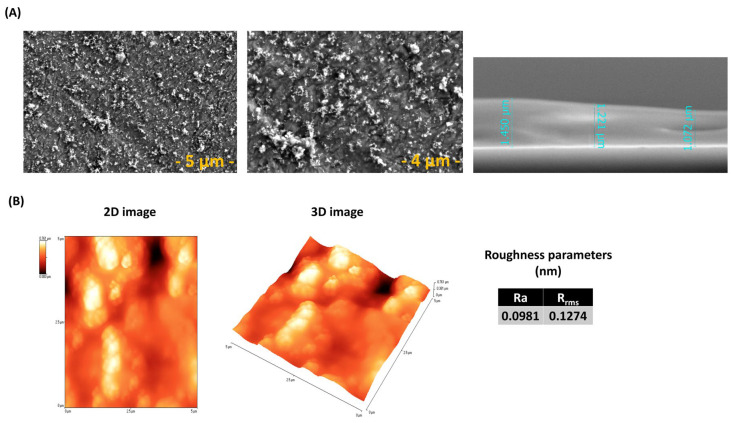
(**A**) Top view SEM images at different magnifications and cross-section SEM micrograph and (**B**) AFM images of BG57 + 0.1LOV/ZnO.

After the MAPLE deposition of BG + 0.1LOV onto ZnO coated SS, the recorded SEM and AFM images highlight the surface morphology and topography of the composite coatings, illustrating their microstructural organization, and the distribution of particulate features across the ZnO underlying layer (Figure 12).

**Figure 13:**
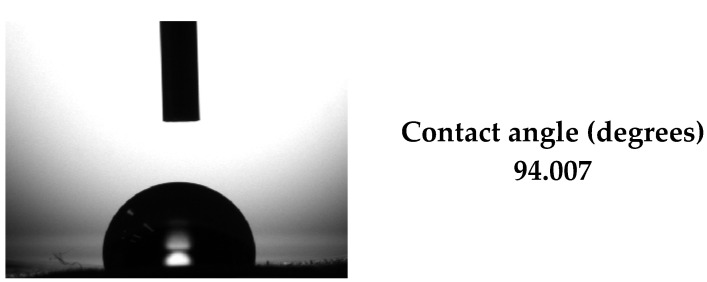
CA of BG57 + 0.1 LOV/ZnO samples.

Figure 13 illustrates the CA measurement of the BG57 + 0.1LOV coating deposited onto ZnO thin films, highlighting the surface wettability characteristics of the composite system.

**Figure 14:**
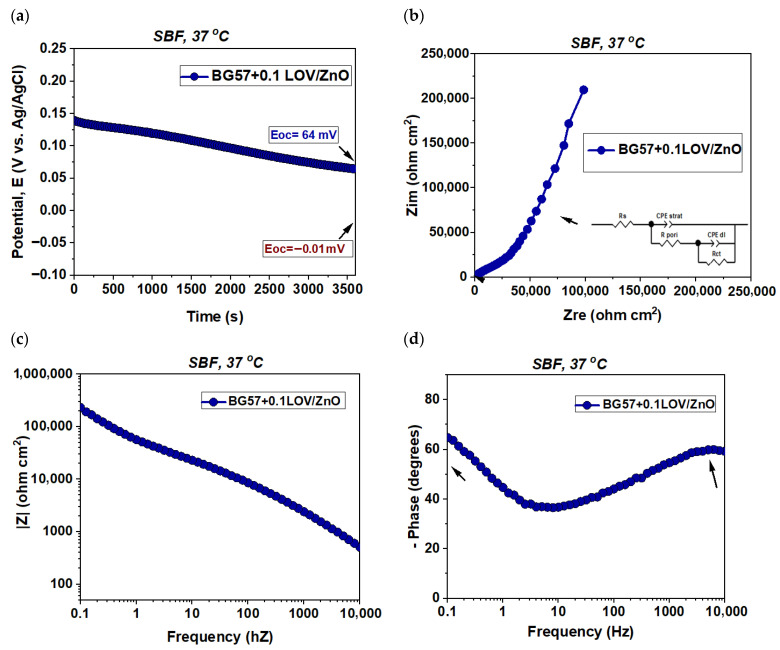
(**a**) Evolution of the open circuit potential over time, (**b**) Nyquist diagram, (**c**) Bode amplitude diagram and (**d**) Bode phase diagram for BG57 + 0.1LOV/ZnO.

The electrochemical performance of the BG57 + 0.1LOV/ZnO multilayer coating was evaluated using OCP monitoring and EIS in SBF at 37 °C. The results presented in Figure 14 and Table 3 provide comprehensive insight into the corrosion resistance of the composite system, highlighting the evolution of the OCP over time as well as the impedance response in both Nyquist and Bode representations.

**Table 3:** Electrochemical parameters determined from the interpretation of the impedance spectra of BG57 + 0.1LOV/ZnO thin films.

	BG57 + 0.1LOV/ZnO
R_s_ (Ω cm^2^)	10
Q_coat_ (μF s^(α−1)^ cm^−2^)	1.14
α__coat_	0.67
R_pore_ (Ω cm^2^)	21,993
Q_dl_ (μFs^(α−1)^ cm^−2^)	5.82
α_dl_	0.57
R_ct_ (Ω cm^2^)	-
*χ* ^2^	1 × 10^−3^

**Figure 15:**
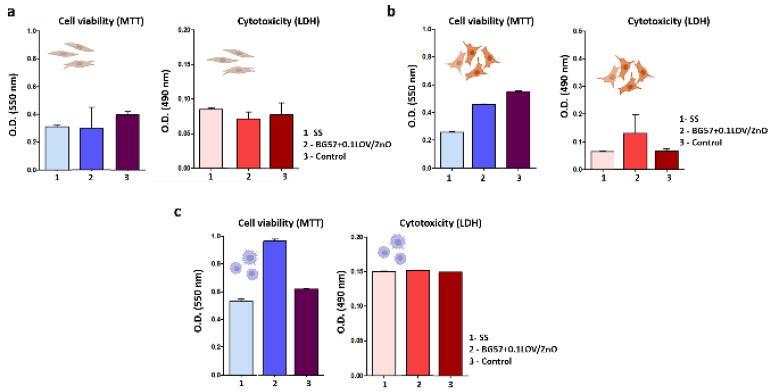
Biocompatibility assessment of the coatings on HDF (**a**), HaCaT (**b**) and RAW (**c**) cells; The control represents cells, steady state conditions, no stimuli. The experiments were performed in triplicate (*n* = 3 independent experiments), and the values shown represent the mean ± standard deviation.

The in vitro biocompatibility of the BG57 + 0.1LOV/ZnO thin films was evaluated using MTT and LDH assays after 24 h of incubation with HDF, HaCaT and RAW cell lines (Figure 15).

**Figure 16:**
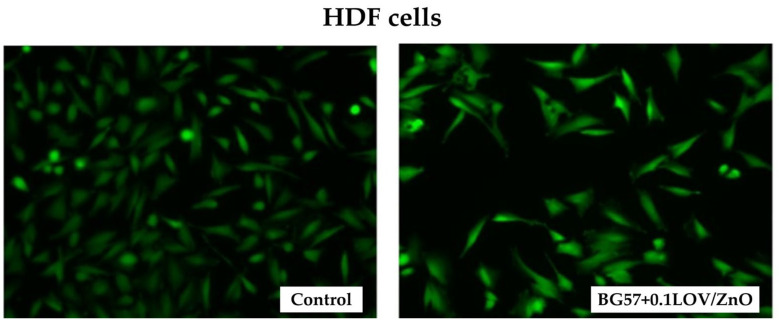
Live/Dead fluorescence images of HDF cells cultured on BG57 + 0.1LOV/ZnO thin films.

Figure 16 shows Live/Dead fluorescence images of HDF cells cultured on BG57 + 0.1LOV/ZnO coatings. The assay highlights viable cells stained green (calcein-AM) and dead cells stained red (ethidium bromide), allowing qualitative assessment of cell viability, morphology, and surface compatibility of the multilayer coating.

**Figure 17:**
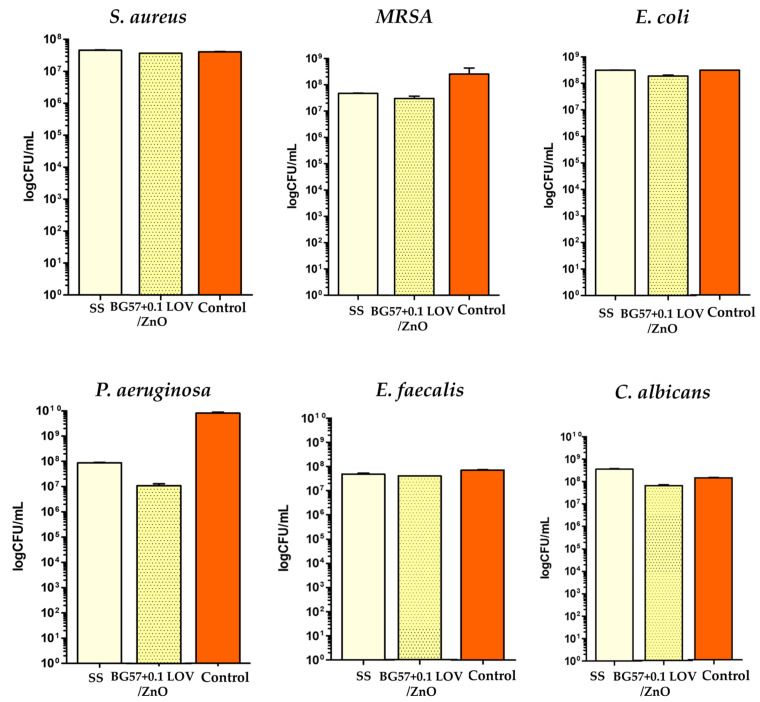
Antimicrobial activity of BG57 + 0.1LOV/ZnO bi-layered structures against *S. aureus*, *MRSA*, *E. coli*, *P. aeruginosa*, *E. faecalis*, *C. albicans*.

The antimicrobial activity of the investigated BG57 + 0.1LOV/ZnO thin films was evaluated by assessing their influence on microbial viability after 24 h of direct contact with standardized bacterial suspensions. The representative images presented below illustrate the differences in microbial survival among the tested samples (Figure 17).

**Figure 18:**
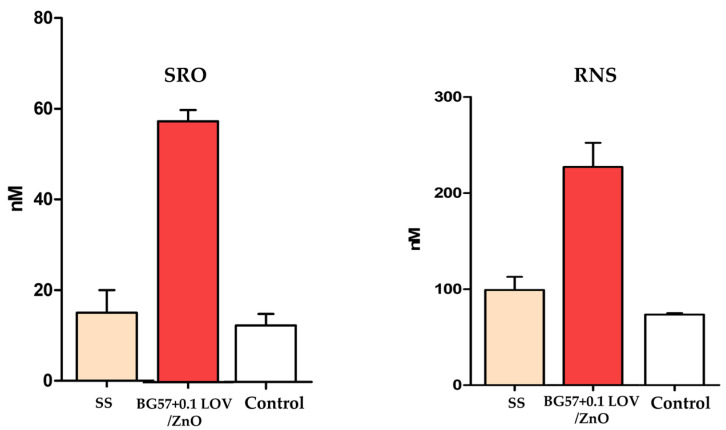
Quantification of SRO (left) and RNS (right) of the BG57 + 0.1LOV/ZnO coatings in the presence of *P. aeruginosa*.

To further elucidate the mechanism underlying the antimicrobial activity of the ZnO-functionalized coatings, the generation of ROS and RNS was quantitatively assessed. The results presented in Figure 18 illustrate the oxidative and nitrosative responses associated with the tested materials, providing insight into their contribution to microbicidal activity.

## 4. Discussion

### 4.1. ZnO Base-Layer Synthesized onto SS by PLD

The SEM micrographs of the PLD-deposited ZnO thin film (Figure 1A) reveal a continuous and compact surface morphology, without visible cracks or delamination, indicating good film integrity. At lower magnification, the surface appears relatively uniform, while higher-magnification images highlight a granular and densely packed microstructure, characteristic of ZnO films grown by PLD. The presence of closely packed grains and interconnected features suggests effective nucleation and growth during deposition, leading to a homogeneous coating [56]. The ZnO layer exhibited a thickness ranging from ~94 to 101 nm, measured at different positions along the cross-section. The relatively narrow variation (~7 nm) confirms good thickness homogeneity. A compact and continuous coating with a well-defined interface was observed, without evidence of cracks or delamination.

The observed micro- to nanoscale surface texturing is consistent with the AFM results and contributes to the measured surface roughness. Such morphology is typical for PLD-grown ZnO layers and is considered advantageous, as it increases the effective surface area while maintaining film continuity. This structural compactness is beneficial for corrosion protection as it limits direct electrolyte access to the underlying substrate [12,57].

Beyond corrosion protection, nanoscale roughness of ZnO films enhances mechanical interlocking and interfacial adhesion of subsequently deposited layers. Studies on ZnO interlayers have demonstrated that controlled surface roughness improves adhesion strength and coating stability under wet and corrosive conditions [58]. In biomedical multilayer systems, ZnO coatings are frequently used as functional underlayers precisely because their nanostructured surfaces support the deposition of organic, polymeric, or bioactive overlayers without interfacial delamination [59].

In hybrid laser-fabricated systems, ZnO layers deposited by PLD provide a chemically stable, corrosion-resistant, and mechanically robust platform, while their nanoscale roughness promotes adhesion and uniform coverage of MAPLE-deposited bioactive coatings. Such architectures can combine improved electrochemical stability with enhanced biological functionality, making ZnO an effective intermediate layer in multilayer biomedical coating [60].

Recorded EDS (Figure 2) spectra exhibited pronounced signals corresponding to the characteristic Zn and O lines, confirming the successful deposition of a ZnO layer on the selected substrate. In addition, Fe and Cr signals were detected, which were found to be consistent with the underlying SS substrate. O can originate from both the native passive oxide film of SS and the deposited ZnO coating.

In the case of the bare SS sample (Figure 3, row 1), although only a single maximum is observed in the Bode phase plot, the Nyquist diagram reveals the presence of two semicircles, indicating the formation of an oxide layer with a compact structure, as suggested by the high values of the layer constant-phase element exponent (α__coat_) in Table 1. The pore resistance of the oxide layer (R_pore_) exhibits a relatively high value after only 6 h of immersion (2163 Ω·cm^2^), followed by a slight increase over time. However, after 48 h, the pore resistance decreases to 1815 Ω·cm^2^, indicating a reduction in corrosion resistance during prolonged exposure. This behavior can be attributed to the presence of chloride ions (Cl^−^) in the SBF, which may destabilize the passive oxide layer formed on the SS surface in contact with biological fluids.

The Bode phase plot (Figure 3, row 2) for ZnO thin films indicates the presence of two-time constants; therefore, the equivalent electrical circuit shown was selected for fitting the experimental data. A slight decrease in electrochemical performance with increasing immersion time can be observed, as evidenced both by the Bode magnitude plot (Figure 3b, second row) and by the reduction in pore resistance (R_pore_) from 105 Ω·cm^2^ after 6 h of immersion to 81 Ω·cm^2^ after 24 h. The relatively low value of the constant-phase element exponent α (~0.6) at the beginning of the test suggests a non-ideal ZnO surface with a degree of porosity that allows electrolyte penetration toward the substrate. After 24 h of immersion, however, the α__coat_ parameter shows a slight increase, reaching a value of 0.67, indicating an improvement in surface quality.

Importantly, the charge-transfer resistance (R_ct_), which is measurable for bare SS, is no longer detectable for the ZnO-coated samples, indicating effective suppression of electrochemical reactions at the metal–electrolyte interface.

Furthermore, the evolution of the α_coat_ parameter for ZnO coatings, from ~0.60 at early immersion times to ~0.67 after 24 h, suggests a gradual improvement in coating surface quality and interfacial homogeneity during immersion. This behavior may be associated with partial pore blocking or surface stabilization effects in the ZnO layer, contributing to its sustained protective function.

The ZnO coatings act as an effective physical and electrochemical barrier, limiting electrolyte access to the SS substrate and mitigating corrosion processes over time. These findings demonstrate that PLD-deposited ZnO layers provide enhanced corrosion protection for SS in physiological environments compared to the native passive oxide layer alone.

### 4.2. BG57 + LOV Single Layers Deposited on Bare SS by MAPLE

The FTIR spectra of the BG57 + LOV thin films, compared to the drop-cast samples, are presented in Figure 4. The spectra exhibited characteristic LOV absorption bands at 3540 cm^−1^ (O–H stretching vibration), 2965 cm^−1^ (asymmetric stretching of the methyl group), 2926 cm^−1^ (asymmetric stretching of the methylene group), and at 1719 and 1683 cm^−1^ corresponding to lactone and ester carbonyl stretching vibrations. Additional bands were observed at 1463 cm^−1^ (asymmetric bending of the methyl group), 1361 cm^−1^ (symmetric bending vibrations of the methyl group), and 1050 cm^−1^, attributed to symmetric stretching vibrations of ester C–O–C bonds. The absorption peak at 1050 cm^−1^ partially overlaps with the characteristic Si–O–Si band of the BG matrix. Furthermore, the absorption band observed at approximately 480 cm^−1^ is assigned to the PO_4_^3−^ group [61].

SEM images of the BG57 + 0.1 LOV thin films (Figure 5A) show a continuous yet moderately heterogeneous surface morphology. At low magnification, the coating appears uniformly distributed without visible cracks or delamination, indicating good structural integrity. Higher magnifications reveal a microstructured surface with dispersed bright particulates, likely corresponding to localized BG57-rich regions. The films exhibit moderate roughness, characteristic of MAPLE-deposited organic–inorganic composites [62], which may enhance surface area for protein adsorption and drug-release behavior [51,63].

In contrast, the BG57 + 0.5 LOV (Figure 5A) coatings display a more heterogeneous morphology. Although film continuity is preserved, higher magnifications reveal larger agglomerated domains and clustered features, suggesting partial phase separation and lovastatin-rich accumulations. AFM analysis and roughness parameters (Figure 5B) further confirm the increased surface roughness and microstructural heterogeneity at higher drug loading.

Increasing LOV concentration from 0.01 to 0.005 leads to reduced morphological homogeneity and greater surface irregularity in MAPLE-deposited BG57-based films, as visible in Figure 5A).

Cross-sectional SEM analysis revealed that the BG57 + 0.1 LOV coating exhibited a thickness of ~229–243 nm, while the BG57 + 0.5 LOV sample showed a thickness of ~357–365 nm. This thickness variation across the measured regions confirms good coating uniformity. A compact and continuous interface with the substrate was observed for both samples, without evidence of interfacial defects.

The measured CA show that the BG + 0.1 LOV film (71.04°) is more hydrophilic than the BG + 0.5 LOV film (80.85°) (Figure 6). Lower CA has been associated with improved protein adsorption and cellular adhesion [64,65], which are often desirable for tissue integration and regeneration applications [66]. The observed increase in CA with higher LOV content suggests that higher organic loading may reduce overall surface polarity or change surface microstructure, leading to slightly reduced wettability. Similar statin-loaded surface coatings have demonstrated significant effects on wettability, where incorporation of simvastatin into collagen matrices reduced the water CA of scaffold surfaces, indicating enhanced hydrophilicity upon drug loading [67]. Because wettability is influenced by both surface chemistry and topography, this difference may also reflect changes in roughness or microstructure associated with higher drug content. This phenomenon has also been explored in other nanostructured coatings where morphology strongly affects wetting behavior [68].

From Figure 7a, it can be observed that the investigated coatings initially exhibited small potential fluctuations and reached a stable value after 1 h of immersion in SBF, the required time depending on each material’s ability to form a more stable oxidized state according to the corrosive environment used. The bare SS substrate recorded the lowest value at the end of the test (E_oc_ = −110 mV), indicating the formation of a corrosion-susceptible surface layer. On the other hand, the addition of LOV into the BG coatings led to an improvement in corrosion resistance, a property that was concentration-dependent. Thus, the most electropositive Eoc value was obtained for BG57 + 0.5LOV (E_oc_ = −49 mV).

The Nyquist plots (Figure 7b) obtained for the investigated materials indicated the presence of incomplete semicircles of different diameters along with a shift in the obtained values toward the high-frequency region. Moreover, the deformation observed in the semicircles is a common characteristic of such materials and is often attributed to surface roughness. Results similar to those described above were observed in the Bode amplitude diagram (Figure 7c), where the impedance modulus (|Z|) recorded higher values for BG57 + 0.5LOV over the applied frequency range, indicating improved corrosion protection of the SS substrate provided by this coating. However, the low impedance phase angle value observed for BG57 + 0.5LOV is characteristic of an accelerated corrosion process.

As previously mentioned, a shift in the obtained values toward the high-frequency region was observed, characteristic of the solution resistance (Table 2). The Rs parameter showed higher values for BG57 + LOV compared to the bare substrate (SS), especially at higher LOV concentrations (R_s_ = 2009 Ω·cm^2^). Considering that the cell geometry and conductivity of the electrolyte remained unchanged throughout the tests, this increase in Rs indicates the presence of degradation products.

A significant increase in pore resistance (R_pore_) was also observed for the coated samples, with slightly higher values depending on the LOV concentration. Although BG57 + 0.5LOV initially appears to provide enhanced protection to the substrate, which is continuously exposed to the electrolyte, it exhibits an accelerated degradation process, as evidenced by the lower charge transfer resistance value (R_ct_ = 12,665 Ω·cm^2^).

The MTT results (Figure 8, left) revealed that the BG57 + 0.1LOV coating exhibited the highest metabolic activity compared to both the control and BG57 + 0.5LOV, indicating enhanced osteoblastic proliferation in the presence of the higher LOV concentration. Although a slight decrease in metabolic activity was observed for BG57 + 0.5LOV relative to the control, all tested samples maintained good cell viability, demonstrating the cytocompatible nature of the coatings.

The LDH assay (Figure 8, right) further confirmed these findings, as both LOV-containing coatings showed lower LDH release compared to the control, with BG57 + 0.1LOV presenting the lowest cytotoxicity levels. The reduced LDH activity indicates preserved membrane integrity and minimal cellular damage.

The Live/Dead fluorescence images (Figure 9) clearly confirm the cytocompatibility of the BG57-based thin films incorporating LOV. For BG57 + 0.1LOV, the cell density appears comparable to or slightly higher than the control, with well-spread cells exhibiting normal morphology and strong green fluorescence, suggesting good adhesion and viability. Notably, the BG57 + 0.5 LOV coating also supports a high number of viable cells, with uniform distribution and minimal red fluorescence. The limited presence of red-stained cells across all groups indicates low cytotoxicity and preserved membrane integrity.

The oxidative stress response induced by the BG57-based coatings was evaluated by quantifying ROS and RNS. As shown in Figure 10, ROS levels in RAW macrophages remained comparable to the control group for both BG57 + 0.5LOV and BG57 + 0.1LOV samples. Although a slight increase in DCFDA fluorescence can be observed for the LOV-containing coatings, the differences are minimal and fall within a close range to the control values, indicating that the materials do not induce significant oxidative stress.

Similarly, nitric oxide production assessed in osteoblasts after 24 h of exposure did not show a pronounced increase compared to the control. The RNS levels remained relatively stable across all tested groups, with only minor variations. Importantly, none of the BG57-based materials triggered an elevated nitrosative response, suggesting the absence of inflammatory activation under the tested conditions.

The antimicrobial performance of the BG57-based coatings incorporating lovastatin was evaluated against representative Gram-positive (*S. aureus*), Gram-negative (*E. coli*, *P. aeruginosa*), and fungal (*C. albicans*) strains (Figure 11).

The antimicrobial behavior of the BG57 coatings incorporating two different lovastatin concentrations (0.1 and 0.5) revealed a strain-dependent response. The optical density (OD_550_) values indicate that the biological effect of LOV incorporation is not uniformly concentration-dependent across all tested microorganisms.

For *S. aureus* and *C. albicans*, the higher LOV concentration (BG57 + 0.5LOV) exhibited higher OD values compared to BG57 + 0.1LOV, indicating reduced antimicrobial efficacy at increased loading. The reduced antimicrobial efficacy of BG57 + 0.5LOV against *S. aureus* and *C. albicans* can be correlated with the morphological and wettability changes induced by higher LOV loading. SEM and AFM analyses revealed that increasing LOV concentration from 0.1 to 0.5 leads to greater surface heterogeneity, larger agglomerates, and increased roughness. While moderate roughness and micro-porosity (BG57 + 0.1LOV) may favor controlled drug release and beneficial surface interactions, excessive aggregation at higher loading may reduce coating compactness and create microenvironments that support microbial adhesion. CA measurements further showed that BG57 + 0.1LOV is more hydrophilic (71.04°) than BG57 + 0.5LOV (80.85°). The reduced wettability at higher LOV content suggests altered surface polarity and interfacial behavior, which may facilitate microbial attachment [56]. Additionally, LOV-rich agglomerates in BG57 + 0.5LOV may affect release kinetics, limiting the immediate availability of the active compound at the coating–microbe interface despite the higher nominal drug concentration.

In contrast, a different trend was observed for Gram-negative strains. For *E.coli* and *P. aeruginosa*, BG57 + 0.5LOV demonstrated lower OD values compared to BG57 + 0.1LOV, indicating enhanced antibacterial activity at the higher LOV concentration. This suggests that Gram-negative bacteria may be more sensitive to increased local concentrations of statins, potentially due to interactions with membrane-associated metabolic pathways or increased disruption of cellular homeostasis [69].

The strain-dependent response highlights the complex interplay between coating composition, drug concentration, microbial cell wall structure, and surface-mediated effects. Gram-negative bacteria, characterized by their outer membrane and lipopolysaccharide-rich envelope, may respond differently to statin-mediated interference compared to Gram-positive bacteria and fungi [70].

### 4.3. Bilayer BG57 + LOV/ZnO Bi-Layered Structure on SS

Based on all of the above, BG57 + 0.1LOV was selected for deposition onto ZnO thin films due to its superior balance between structural stability, biological performance, and antimicrobial efficacy. Compared to the higher LOV loading, the 0.1LOV formulation exhibits a more homogeneous and compact morphology, moderate roughness, and improved wettability. It also demonstrates high osteoblast viability, low cytotoxicity, and no induction of oxidative stress. Moreover, it maintains effective antimicrobial activity, particularly against *S. aureus*, a clinically relevant pathogen in implant-associated infections. Collectively, these characteristics make BG57 + 0.1LOV the most suitable candidate for developing multifunctional ZnO-based bioactive coatings.

SEM images (Figure 12) show the top morphology of BG57 + 0.1LOV/ZnO. The micrographs reveal the presence of irregularly shaped particulates arbitrarily distributed/scattered across the ZnO surface. The surface appears covered by dispersed clusters and granular features, indicating successful transfer of the organic–inorganic composite material [62]. Such particulate-rich topography is typical for MAPLE processes and reflects the matrix-assisted ejection and deposition mechanism, which often results in microstructured and heterogeneous surface architectures [71]. These structures are beneficial for cells adhesion [72]. Cross-sectional SEM analysis of the BG57 + 0.1 LOV coating deposited onto the ZnO thin film (Figure 12) revealed a thickness ranging from ~1.07 to 1.45 µm. The thickness variation across the examined region indicates acceptable uniformity for the multilayer structure.

The calculated roughness parameters (Ra = 0.0981 µm and Rrms = 0.1274 µm) (Figure 12) indicate moderate surface roughness, which is advantageous for biomedical applications. Such topography can enhance surface area and promote protein adsorption. The roughness values remain within a range that supports cellular adhesion without excessively favoring bacterial colonization [73].

Figure 13 shows that the BG57 + 0.1LOV/ZnO sample exhibits a CA of 94.00°, indicating a more hydrophobic surface compared to BG57 + 0.1LOV deposited directly on SS (71.04°) and BG57 + 0.5LOV on SS (80.85°) (Figure 6). While the BG57-based coatings on bare SS displayed relatively good hydrophilicity, particularly at the lower LOV concentration, the introduction of the ZnO interlayer significantly increases the contact angle, suggesting altered surface chemistry and/or topography.

This increase in CA for the BG57 + 0.1LOV/ZnO system may result from the combined influence of ZnO surface characteristics and the MAPLE-deposited composite morphology. Since wettability depends on both chemical composition and surface roughness [68], the presence of the ZnO layer likely modifies interfacial energy and microstructural organization, leading to reduced surface polarity and lower wettability [74,75,76].

Therefore, compared to BG57 coatings deposited on bare SS, the BG57 + 0.1LOV/ZnO system demonstrates a shift toward increased hydrophobicity. While moderate hydrophilicity is generally advantageous for cellular interactions, the slightly higher CA in the ZnO-containing system may also contribute to altered bacterial adhesion behavior, highlighting the importance of interlayer effects in multifunctional coating design [76].

Following immersion in SBF at 37 °C, all investigated materials reached a constant potential after approximately 1 h, indicating stabilization of the material–electrolyte interface (Figure 14a). The BG57 + 0.1LOV/ZnO sample exhibited improved corrosion resistance, as reflected by its electropositive E_oc_ value of +64 mV, suggesting the formation of a steady and protective surface layer in the simulated physiological environment.

The Nyquist plots obtained for the BG57 + 0.1LOV/ZnO coatings (Figure 14b) revealed the presence of incomplete semicircles with different diameters, indicating distinct electrochemical responses at the material–electrolyte interface. In agreement with the Eoc evolution results, the Bode amplitude diagram (Figure 14c) reflects variations in corrosion resistance. Analysis of the Bode phase diagram (Figure 14d) for the BG57 + 0.1LOV/ZnO coating revealed the presence of two incomplete phase maxima, indicated by arrows, located in the high- and low-frequency regions. This behavior suggests the occurrence of two-time constants, typically associated with processes occurring at the top/surface? layer and at the coating/substrate interface. The presence of these features indicates a more complex electrochemical behavior and may reflect an accelerated corrosion process compared to single-time-constant systems.

To better highlight the observed time constants, the impedance data were fitted using the equivalent electrical circuit presented in Figure 14b. The constant phase element (CPE) parameters indicated α_coat_ = 0.67 and α_dl_ = 0.57, suggesting deviation from ideal capacitive behavior, likely due to surface heterogeneity and non-uniform current distribution.

A comparative analysis of the electrochemical performance reveals progressive improvements from bare SS to the multilayer BG57 + 0.1LOV/ZnO system.

In the case of bare SS (Figure 3, row 1), the Nyquist plot shows two semicircles, despite the presence of a single maximum in the Bode phase diagram, indicating the formation of a native oxide layer with relatively compact characteristics. Initially, the oxide layer exhibits moderate pore resistance (Rpore ≈ 2163 Ω·cm^2^ after 6 h), but prolonged immersion leads to a decrease (≈1815 Ω·cm^2^ after 48 h), suggesting destabilization of the passive film due to chloride ions in SBF. The presence of a measurable charge-transfer resistance (Rct) confirms ongoing electrochemical reactions at the substrate–electrolyte interface. For ZnO-coated samples (Figure 3, row 2), two time constants are clearly observed, corresponding to processes within the ZnO layer and at the ZnO/SS interface. Although initial pore resistance values are relatively low (105 Ω·cm^2^ at 6 h), the gradual increase in the α_coat_ parameter from ~0.60 to ~0.67 suggests progressive surface stabilization and improved interfacial homogeneity. Importantly, the disappearance of R_ct_ compared to bare SS indicates effective suppression of direct electrochemical reactions at the substrate interface, confirming the protective role of ZnO.

In the case of BG57 + LOV coatings deposited directly on SS (Figure 7), incomplete semicircles and deformation in Nyquist plots reflect surface roughness and coating heterogeneity. The Bode amplitude plots show increased impedance modulus values, particularly for BG57 + 0.5LOV, indicating improved initial corrosion resistance compared to bare SS. However, the reduced phase angle values and lower R_ct_ (12,665 Ω·cm^2^ for BG57 + 0.5LOV) suggest an accelerated degradation despite the improved barrier effect. The increase in solution resistance (R_s_) further indicates the accumulation of degradation products in the electrolyte.

The BG57 + 0.1LOV/ZnO multilayer system (Figure 14) exhibits a more complex electrochemical response characterized by two distinct time constants in the Bode phase plot, corresponding to the composite coating layer and the coating/substrate interface. The extracted CPE parameters (α_coat_ = 0.67 and α_dl_ = 0.57) indicate non-ideal capacitive behavior due to surface heterogeneity, but remain comparable to stabilized ZnO surfaces. Compared to single-layer coatings, the multilayer configuration benefits from ZnO-mediated suppression of interfacial charge transfer while incorporating the bioactive characteristics of the BG57 + 0.1LOV layer.

The improved corrosion resistance observed for the BG57 + 0.1LOV/ZnO multilayer coating can be attributed to the combined effect of the individual layers, each contributing through different but complementary protection mechanisms. The ZnO base layer deposited by PLD acts as the main corrosion-protective barrier. Due to its compact and continuous morphology, confirmed by SEM and AFM analyses, it limits the ingress of the electrolyte toward the substrate and helps stabilize the metal-electrolyte interface. On the other hand, the BG57 + 0.1LOV layer deposited by MAPLE provides an additional protective function. The BG matrix can partially protect the ZnO layer from direct exposure to the electrolyte and provides bioactive functionality. In addition, the moderately rough microstructured morphology of the MAPLE-deposited coating increases surface coverage and may facilitate the formation of a stable surface layer when the material is immersed in SBF. The obtained results suggest that combining a corrosion-resistant oxide interlayer with a bioactive top coating improves the stability of the SS substrate under physiological conditions.

For the HDF cell line (Figure 15a), the MTT assay shows that BG57 + 0.1LOV/ZnO maintains metabolic activity at levels comparable to bare SS and slightly below the control. Importantly, LDH values for BG57 + 0.1LOV/ZnO are lower than those observed for SS and close to the control group, indicating minimal membrane damage. These results confirm good cytocompatibility of the multilayer coating toward fibroblasts, supporting its suitability for hard-tissue interface applications. In the case of HaCaT cells (Figure 15b), BG57 + 0.1LOV/ZnO demonstrates significantly improved metabolic activity compared to SS, although it is slightly lower than the control. The LDH values remain within a moderate range, showing no marked cytotoxic effect. The increased MTT signal relative to SS suggests that the composite coating enhances epithelial cell viability, likely due to improved surface chemistry and wettability provided by the BG57 + 0.1LOV layer. For RAW cells (Figure 15b), BG57 + 0.1LOV/ZnO exhibits the highest metabolic activity among the tested samples, exceeding both SS and control in the MTT assay. This indicates strong cellular activation or proliferation. Importantly, LDH values remain comparable to SS and control, suggesting that the increased metabolic activity is not associated with a cytotoxic effects. The absence of elevated LDH release indicates preserved membrane integrity and no inflammatory cytotoxic response. The higher variation observed for the BG57 + 0.1LOV/ZnO coating may be attributed to biological variability in the cellular response, which can occur due to differences in cell metabolic activity and sensitivity to surface-derived factors such as ion release or ROS generation. Despite this variability, the overall trend of the results remained consistent across independent experiments, demonstrating good reproducibility of the biological response.

The Live/Dead fluorescence images of HD cells revealed (Figure 16) high viability across all tested surfaces (Control, and BG57 + 0.1LOV/ZnO). The cells exhibit the typical elongated, spindle-shaped fibroblast morphology, suggesting good adhesion and spreading. Compared to Control, the BG57 + 0.1LOV/ZnO surface supports a comparable cell density and preserved cellular morphology, without noticeable red fluorescence which would indicate membrane damage. As expected, the control sample shows the highest cell density, while BG57 + 0.1LOV/ZnO sustains a healthy and well-distributed cell population.

The antimicrobial activity of the BG57 + 0.1LOV/ZnO coating was evaluated against a panel of clinically relevant Gram-positive, Gram-negative, and fungal strains (Figure 17) to determine its spectrum of activity and strain-dependent behavior. The BG57 + 0.1LOV/ZnO coating shows a slight reduction in CFU compared to SS and control, but the difference appears modest (less than 1 log). This suggests a limited antibacterial effect against *S. aureus*. While some inhibitory activity is present, the bi-layered structure is not strongly bactericidal under the tested conditions. For MRSA, BG57 + 0.1LOV/ZnO demonstrates a clearer microbe reduction compared to the control and SS. The decrease appears more pronounced than in the case of common *S. aureus*, indicating enhanced activity against MRSA. This suggests a beneficial effect of the LOV/ZnO combination against antibiotic-resistant Gram-positive bacteria. The antimicrobial effect against *E. coli* appears minimal. CFU values for BG57 + 0.1LOV/ZnO are close to those of SS and control, indicating limited activity against the Gram-negative strain. The outer membrane characteristic of Gram-negative bacteria may reduce susceptibility. A marked reduction in CFU is observed for BG57 + 0.1LOV/ZnO compared to the control, with approximately a 2–3 log decrease in the case of *P. aeruginosa*. This indicates significant antibacterial activity against *P. aeruginosa*, a clinically relevant and biofilm-forming Gram-negative pathogen. The ZnO component likely contributes strongly to this effect. Only minor differences are observed between BG57 + 0.1LOV/ZnO, SS, and control and the coating does not appear to significantly inhibit *E. faecalis* growth under the tested conditions. In the case of *C. albicans*, the coating shows a noticeable reduction in fungal CFU compared to SS and control (approximately 1 log). This suggests moderate antifungal activity, which is, however, relevant for preventing opportunistic infections.

The antimicrobial performance appears strain-dependent, with enhanced efficacy particularly against resistant Gram-positive and selected Gram-negative (*P. aeruginosa)* strains. The results suggest a synergistic contribution of ZnO (membrane disruption, ROS generation) and LOV (metabolic interference), leading to selective antimicrobial behavior.

The BG57 + 0.1LOV/ZnO sample shows markedly increased ROS production (~58 nM) compared to SS and control (Figure 18, left). This significant elevation indicates that the ZnO-containing coating strongly promotes oxidative stress at the microbial interface. The control and SS samples exhibit relatively low and comparable ROS levels, suggesting minimal intrinsic oxidative activity. The enhanced ROS generation in the BG57 + 0.1LOV/ZnO system supports the hypothesis that ZnO contributes to antimicrobial activity through oxidative mechanisms, likely involving superoxide radicals and hydrogen peroxide formation. In addition to ROS production at the material surface, the ZnO film may further enhance antibacterial activity through the release of Zn^2+^ ions and direct interactions with bacterial membranes. These interactions increase membrane permeability and promote the leakage of intracellular components, ultimately leading to bacterial cell damage. The combination of oxidative stress, ionic release, and membrane disruption results in a multifactorial antimicrobial mechanism, which significantly enhances antibacterial efficiency while reducing the likelihood of bacterial resistance development.

Similarly, RNS production is substantially higher for BG57 + 0.1LOV/ZnO compared to SS and control (Figure 18, right). The pronounced increase in nitrosative species suggests that the coating induces additional nitrosative stress, which can contribute to microbial membrane damage, protein nitrosylation, and disruption of metabolic pathways. The moderate RNS levels observed for SS may reflect baseline interfacial reactions, while the control maintains the lowest nitrosative response.

ROS and RNS are known to exert strong antimicrobial effects by inducing oxidative stress in bacterial cells, leading to damage of lipids, proteins, and nucleic acids. Because ROS target multiple cellular components simultaneously, they can effectively kill bacteria and reduce the likelihood of resistance development [77].

However, mammalian cells generally tolerate moderate ROS levels better than bacteria due to their more advanced antioxidant defense systems, including enzymes such as superoxide dismutase, catalase, and glutathione peroxidase, as well as intracellular antioxidants like glutathione that maintain redox homeostasis [78].

Furthermore, structural and physiological differences between bacterial and mammalian cells contribute to this selectivity. Bacterial membranes are more vulnerable to oxidative damage, while mammalian cells can internalize particles and buffer oxidative stress more efficiently, which reduces cytotoxicity while maintaining antibacterial activity.

## 5. Conclusions

In this study, a multifunctional multilayer coating based on ZnO and BG57-LOV was efficaciously fabricated on SS substrates using combined PLD and MAPLE approaches. The strategy aimed to integrate corrosion protection, bioactivity, and antimicrobial performance into a single architectured surface system. The ZnO base layer provided effective corrosion protection and served as a stable interfacial barrier, while the BG57 + LOV top layer preserved the chemical integrity of the bioactive components and exhibited favorable surface morphology and wettability. Among the tested formulations, BG57 + 0.1LOV demonstrated the most balanced performance in terms of cytocompatibility, corrosion behavior, and antimicrobial activity, and was therefore selected for the multilayer system. The resulting BG57 + 0.1LOV/ZnO coating showed improved electrochemical stability, excellent cytocompatibility with fibroblasts, keratinocytes, and macrophages, and selective antimicrobial activity against clinically relevant pathogens.

Overall, the BG57 + 0.1LOV/ZnO multilayer coating represents a promising multifunctional system for implant-associated biomedical applications.

## Figures and Tables

**Table 1:** Electrochemical parameters determined from the interpretation of the impedance spectra of bare SS and ZnO thin films recorded after 6, 12, 18, and 24 h of immersion in SBF at 37 °C (solution resistance—R_s_, coating capacitance—Q_coat_, pore resistance—R_pore_, double layer capacitance—Q_dl_, charge transfer resistance—R_ct,_ exponent used to calculate the impedance of the constant phase element associated with the thin film—α_strat_, exponent used to calculate the impedance of the constant phase element associated with the electrical double layer—α_dl_, statistical parameter that describes the goodness of fit between the simulated model and the experimental data—*χ*^2^).

Experimental Specimen	SS	ZnO	SS	ZnO	SS	ZnO	SS	ZnO
	6 h		12 h		18 h		24 h	
R_s_ (Ω cm^2^)	40.21	31.59	38.25	30.53	36.27	31.23	34.43	34.19
Q_coat_ (μF s^(α−1)^ cm^−2^)	31.97	146.21	32.13	174.71	33.19	103.86	28.52	95.98
α_strat_	0.82	0.61	0.83	0.60	0.83	0.65	0.83	0.67
*R*_ore_ (Ω cm^2^)	2163	105	2247	104	2224	85	1815	81
*Q*_dl_ (μF s^(α−1)^ cm^−2^)	865.51	598.94	871.15	666.46	886.01	799.49	865.86	891.63
α_dl_	1	0.60	1	0.62	1	0.61	0.96	0.62
*R*_ct_ (Ω cm^2^)	1639	-	1660	-	1687	-	1771	-
*χ* ^2^	9 × 10^4^	2 × 10^4^	1 × 10^3^	2 × 10^4^	1 × 10^3^	1 × 10^4^	9 × 10^4^	7 × 10^5^

**Table 2:** Parameters obtained from fitting the EIS curves after 1 h immersion in SBF.

	SS	BG57 + 0.01LOV	BG57 + 0.5LOV
R_s_ (Ω cm^2^)	198	378	2009
Q_coat_ (μF s^(α−1)^ cm^−2^)	215.61	37.38	0.67
α__coat_	0.54	0.51	0.57
R_pore_ (Ω cm^2^)	1267	2193	2210
Q_d_*_l_* (μFs^(α−1)^ cm^−2^)	66.39	360.00	147.55
α__dl_	0.85	0.39	0.54
R_ct_ (Ω cm^2^)	-	110,880	12,665
χ^2^	7 × 10^−4^	3 × 10^−4^	1 × 10^−4^

## Data Availability

The original contributions presented in the study are included in the article; further inquiries can be directed to the corresponding author.
